# 4-[(4-Benzyl­oxybenzyl­idene)amino]-1,5-dimethyl-2-phenyl-1*H*-pyrazol-3(2*H*)-one

**DOI:** 10.1107/S1600536812014262

**Published:** 2012-04-06

**Authors:** Grzegorz Dutkiewicz, Divya N. Shetty, B. Narayana, H. S. Yathirajan, Maciej Kubicki

**Affiliations:** a Department of Chemistry, Adam Mickiewicz University, Grunwaldzka 6, 60-780 Poznań, Poland; b Department of Studies in Chemistry, Mangalore University, Mangalagangotri-574 199, India; c Department of Studies in Chemistry, University of Mysore, Mysore 570 006, India

## Abstract

In the title mol­ecule, C_25_H_23_N_3_O_2_, two terminal phenyl rings are twisted by 50.20 (6) and 71.26 (5)° from the mean plane (r.m.s. deviation = 0.032 Å) of the central benzyl­idene–amino–pyrazolone fragment. The N atoms of the pyrazole ring have a pyramidal environment, the sums of the valence angles around them being 353.5 (2) and 347.3 (2)°. The crystal structure is stabilized by C—H⋯O interactions.

## Related literature

Related crystal structures have been described by Shi (2005 ▶), Jun (2005 ▶), Zhen *et al.* (2006 ▶), Liu *et al.* (2006 ▶), Diao & Chen (2006 ▶), Duan *et al.* (2006 ▶), Hu (2006 ▶) and Zhang *et al.* (2006 ▶).
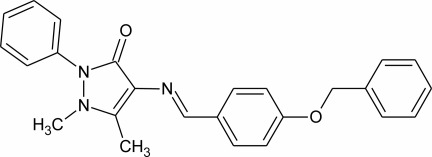


## Experimental

### 

#### Crystal data

C_25_H_23_N_3_O_2_
*M* *_r_* = 397.46
Monoclinic, 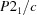

*a* = 19.8137 (19) Å
*b* = 6.1588 (4) Å
*c* = 18.0784 (14) Å
β = 108.881 (9)°
*V* = 2087.4 (3) Å^3^
*Z* = 4
Mo *K*α radiation
μ = 0.08 mm^−1^
*T* = 295 K
0.5 × 0.4 × 0.2 mm

#### Data collection

Agilent Xcalibur Eos diffractometer
Absorption correction: multi-scan (*CrysAlis PRO*; Agilent, 2011 ▶) *T*_min_ = 0.890, *T*_max_ = 1.000
8408 measured reflections
4352 independent reflections
3298 reflections with *I* > 2σ(*I*)
*R*_int_ = 0.018

#### Refinement

*R*[*F* ^2^ > 2σ(*F* ^2^)] = 0.052
*wR*(*F* ^2^) = 0.119
*S* = 1.04
4352 reflections
363 parameters
All H-atom parameters refined
Δρ_max_ = 0.68 e Å^−3^
Δρ_min_ = −0.22 e Å^−3^

### 

Data collection: *CrysAlis PRO* (Agilent, 2011 ▶); cell refinement: *CrysAlis PRO*; data reduction: *CrysAlis PRO*; program(s) used to solve structure: *SIR92* (Altomare *et al.*, 1993 ▶); program(s) used to refine structure: *SHELXL97* (Sheldrick, 2008 ▶); molecular graphics: *XP* in *SHELXTL* (Sheldrick, 2008 ▶); software used to prepare material for publication: *SHELXL97*.

## Supplementary Material

Crystal structure: contains datablock(s) I, global. DOI: 10.1107/S1600536812014262/cv5278sup1.cif

Structure factors: contains datablock(s) I. DOI: 10.1107/S1600536812014262/cv5278Isup2.hkl

Supplementary material file. DOI: 10.1107/S1600536812014262/cv5278Isup3.cml

Additional supplementary materials:  crystallographic information; 3D view; checkCIF report

## Figures and Tables

**Table 1:** Hydrogen-bond geometry (Å, °)

*D*—H⋯*A*	*D*—H	H⋯*A*	*D*⋯*A*	*D*—H⋯*A*
C16—H16⋯O49^i^	0.990 (18)	2.486 (18)	3.470 (2)	172.6 (15)
C21—H21*C*⋯O5^ii^	0.97 (3)	2.58 (3)	3.352 (3)	137.3 (19)
C31—H31*A*⋯O5^ii^	0.97 (2)	2.65 (2)	3.517 (3)	149.1 (17)
C52—H52⋯O5^iii^	0.96 (3)	2.51 (3)	3.411 (3)	156 (2)

Symmetry codes: (i) 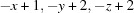; (ii) 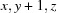; (iii) 
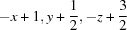
.

**Fig. 1.**
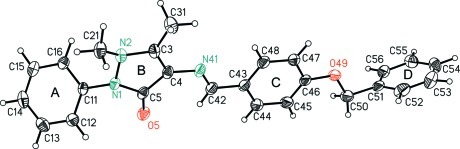
The molecular structure of 1 showing the atom labelling scheme and ring labels (cf. Comment). Displacement ellipsoids are drawn at the 50% probability level, hydrogen atoms are depicted as spheres with arbitrary radii.

**Fig. 2.**
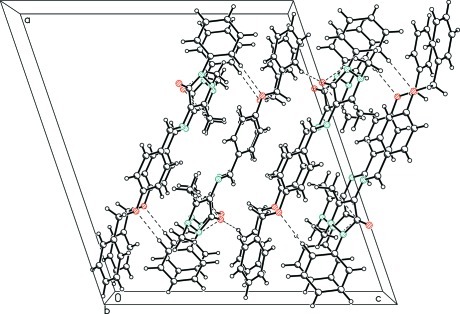
A portion of the crystal packing viewed along [010] direction; C—H···O hydrogen bonds (cf. Table 1) are shown as dashed lines.

## supplementary crystallographic information

### Comment

Pyrazolone, as a prominent structural motif, is found in numerous pharmaceutically active compounds. Due to the easy preparation and rich biological activity, pyrazolone framework plays an essential role and represents an interesting template for combinatorial and medicinal chemistry. Indeed, pyrazolone based derivatives have shown several biological activities such as analgesic, anti-inflammatory, antipyretic, antiarrhythmic, antifungal, muscle relaxing, psychoanaleptic, anticonvulsant, enzyme inhibiting, antidiabetic and antibacterial activities. So, the chemistry of pyrazolone and its derivatives is particularly interesting because of their potential application in medicinal chemistry. Here we present the results of the X-ray structure determination of the title compound, 1.

Quite recently, the crystal structures of a series of similar compounds, with substituted rings C and D (*cf.* Fig. 1) have been reported, namely, 3-methoxy (*C*),2,4-dichloro (D) (Zhen *et al.*, 2006); 2,4-dichloro (D) (Liu *et al.*, 2006); 3-methoxy (C), 4-nitro (D) (Diao & Chen, 2006); 3-methoxy (C), 4-chloro (D) (Duan *et al.*, 2006); 4-chloro (D) (Hu, 2006); 3-ethoxy (*C*),4-chloro (D) (Zhang *et al.*, 2006); 3-methoxy (C) (Shi, 2005), and 3-ethoxy (C) (Jun, 2005).

Compound 1, without additional substituents on the phenyl rings, might be regarded as the reference molecule. It has almost perfectly coplanar central part, consisting of two rings B and C (pyrazolone and phenyl) and the linking C—N=C—C chain (maximum deviation from the least-squares plane is 0.070 (1) Å). The dihedral angle between the planes of the two rings B and C is only 1.42 (13)°, and is significantly smaller than in the other similar molecules (6.21 (10)° - 39.24 (5)°). The overall conformation of the molecule might be described either by the dihedral angle between the planes of terminal phenyl rings (62.71 (6)°) or by the dihedral angles between the central plane and terminal ring planes (50.20 (6)° with ring A, 71.26 (5)° with ring D). These last values are generally consistent with those referred for similar compounds (48.05 (6)° - 72.97 (8)° for A, 39.49 (14)° - 86.16 (7)° for D).

In the pyrazolone ring, the N atoms of the pyrazole ring have pyramidal environment, sums of the valence angles around them are 353.5° for N1 and 347.3° for N2. The bond lengths pattern within this ring suggests significant delocalization and is also typical for these compounds, in contrast, the bond N41—C42 (1.276 (2) Å) has an obvious double-bond character.

In the crystal structure relatively short and linear C16—H16···O49^i^ hydrogen bonds join molecules into centrosymmetric dimers; these dimers, in turn, by means of other, still weaker C—H···O contacts expand in two dimensions (Table 1, Fig. 2).

### Experimental

The mixture of 4-amino-1,5-dimethyl-2-phenyl-1,2-dihydro-3*H*-pyrazol-3-one (2.03 g, 0.01 mol) and 4-benzyloxybenzaldehyde (2.12 g, 0.01 mol) was refluxed in 30 ml e thanol with two drops of sulfuric acid for 3 h. The crude product obtained was filtered and recrystallized from ethanol. Good quality crystals were obtained by the evaporation of the solution in DMF (m.p: 438 K).

### Refinement

Hydrogen atoms were found in difference Fourier maps and isotropically refined.

### Crystal data

**Table:**

C_25_H_23_N_3_O_2_	*F*(000) = 840
*M**_r_* = 397.46	*D*_x_ = 1.265 Mg m^−^^3^
Monoclinic, *P*2_1_/*c*	Mo *K*α radiation, λ = 0.71073 Å
*a* = 19.8137 (19) Å	Cell parameters from 2304 reflections
*b* = 6.1588 (4) Å	θ = 3.1–28.2°
*c* = 18.0784 (14) Å	µ = 0.08 mm^−^^1^
β = 108.881 (9)°	*T* = 295 K
*V* = 2087.4 (3) Å^3^	Block, yellow
*Z* = 4	0.5 × 0.4 × 0.2 mm

### Data collection

**Table:**

Agilent Xcalibur Eos diffractometer	4352 independent reflections
Radiation source: Enhance (Mo) X-ray Source	3298 reflections with *I* > 2σ(*I*)
Graphite monochromator	*R*_int_ = 0.018
Detector resolution: 16.1544 pixels mm^-1^	θ_max_ = 28.3°, θ_min_ = 3.3°
ω–scan	*h* = −25→26
Absorption correction: multi-scan (*CrysAlis PRO*; Agilent, 2011)	*k* = −8→8
*T*_min_ = 0.890, *T*_max_ = 1.000	*l* = −19→24
8408 measured reflections	

### Refinement

**Table:**

Refinement on *F*^2^	Primary atom site location: structure-invariant direct methods
Least-squares matrix: full	Secondary atom site location: difference Fourier map
*R*[*F*^2^ > 2σ(*F*^2^)] = 0.052	Hydrogen site location: inferred from neighbouring sites
*wR*(*F*^2^) = 0.119	All H-atom parameters refined
*S* = 1.04	*w* = 1/[σ^2^(*F*_o_^2^) + (0.0411*P*)^2^ + 1.0652*P*] where *P* = (*F*_o_^2^ + 2*F*_c_^2^)/3
4352 reflections	(Δ/σ)_max_ = 0.001
363 parameters	Δρ_max_ = 0.68 e Å^−^^3^
0 restraints	Δρ_min_ = −0.22 e Å^−^^3^

### Special details

**Table:**

Geometry. All s.u.'s (except the s.u. in the dihedral angle between two l.s. planes) are estimated using the full covariance matrix. The cell s.u.'s are taken into account individually in the estimation of s.u.'s in distances, angles and torsion angles; correlations between s.u.'s in cell parameters are only used when they are defined by crystal symmetry. An approximate (isotropic) treatment of cell s.u.'s is used for estimating s.u.'s involving l.s. planes.
Refinement. Refinement of *F*^2^ against ALL reflections. The weighted *R*-factor *wR* and goodness of fit *S* are based on *F*^2^, conventional *R*-factors *R* are based on *F*, with *F* set to zero for negative *F*^2^. The threshold expression of *F*^2^ > 2σ(*F*^2^) is used only for calculating *R*-factors(gt) *etc*. and is not relevant to the choice of reflections for refinement. *R*-factors based on *F*^2^ are statistically about twice as large as those based on *F*, and *R*- factors based on ALL data will be even larger.

### Fractional atomic coordinates and isotropic or equivalent isotropic displacement parameters (Å^2^)

**Table:**

	*x*	*y*	*z*	*U*_iso_*/*U*_eq_	
N1	0.77141 (7)	1.0983 (2)	1.10006 (8)	0.0236 (3)	
C11	0.83244 (8)	1.0018 (3)	1.15422 (9)	0.0213 (4)	
C12	0.88429 (10)	0.9158 (3)	1.12682 (11)	0.0278 (4)	
H12	0.8798 (9)	0.924 (3)	1.0744 (11)	0.027 (5)*	
C13	0.94283 (10)	0.8168 (3)	1.17855 (12)	0.0334 (5)	
H13	0.9787 (11)	0.760 (3)	1.1597 (11)	0.037 (6)*	
C14	0.95142 (10)	0.8101 (3)	1.25755 (12)	0.0354 (5)	
H14	0.9920 (11)	0.744 (3)	1.2934 (11)	0.035 (5)*	
C15	0.90004 (10)	0.8993 (3)	1.28475 (11)	0.0310 (4)	
H15	0.9032 (10)	0.893 (3)	1.3398 (12)	0.039 (6)*	
C16	0.83978 (9)	0.9942 (3)	1.23326 (10)	0.0253 (4)	
H16	0.8016 (9)	1.053 (3)	1.2519 (10)	0.024 (5)*	
N2	0.73781 (8)	1.2714 (2)	1.12442 (8)	0.0253 (3)	
C21	0.78145 (11)	1.4594 (3)	1.15796 (13)	0.0324 (5)	
H21A	0.7554 (12)	1.554 (4)	1.1837 (13)	0.052 (7)*	
H21B	0.8227 (12)	1.412 (4)	1.1980 (13)	0.049 (6)*	
H21C	0.7936 (13)	1.536 (4)	1.1174 (16)	0.070 (8)*	
C3	0.67109 (9)	1.2867 (3)	1.07207 (10)	0.0261 (4)	
C31	0.62346 (11)	1.4701 (4)	1.07381 (14)	0.0364 (5)	
H31A	0.6409 (11)	1.606 (4)	1.0604 (12)	0.045 (6)*	
H31B	0.6190 (12)	1.488 (4)	1.1266 (15)	0.059 (7)*	
H31C	0.5748 (13)	1.442 (4)	1.0366 (14)	0.061 (7)*	
C4	0.65791 (9)	1.1164 (3)	1.02127 (10)	0.0258 (4)	
C5	0.72166 (9)	0.9880 (3)	1.03848 (9)	0.0242 (4)	
O5	0.73457 (7)	0.8174 (2)	1.01041 (7)	0.0324 (3)	
N41	0.59094 (8)	1.0843 (3)	0.96662 (8)	0.0289 (4)	
C42	0.57671 (10)	0.9211 (4)	0.92063 (10)	0.0306 (4)	
H42	0.6106 (11)	0.804 (3)	0.9202 (11)	0.038 (6)*	
C43	0.50516 (9)	0.8971 (4)	0.86385 (10)	0.0311 (5)	
C44	0.48958 (11)	0.7305 (4)	0.80983 (11)	0.0407 (5)	
H44	0.5249 (11)	0.619 (4)	0.8114 (12)	0.042 (6)*	
C45	0.42241 (11)	0.7081 (4)	0.75510 (12)	0.0443 (6)	
H45	0.4146 (11)	0.584 (4)	0.7209 (13)	0.047 (6)*	
C46	0.36996 (9)	0.8553 (4)	0.75447 (10)	0.0372 (5)	
C47	0.38462 (10)	1.0237 (4)	0.80834 (11)	0.0363 (5)	
H47	0.3460 (11)	1.129 (3)	0.8060 (11)	0.039 (6)*	
C48	0.45139 (10)	1.0441 (4)	0.86208 (11)	0.0343 (5)	
H48	0.4620 (10)	1.163 (3)	0.8994 (12)	0.036 (6)*	
O49	0.30121 (7)	0.8475 (3)	0.70470 (7)	0.0454 (4)	
C50	0.28438 (11)	0.6850 (5)	0.64450 (13)	0.0495 (7)	
H50A	0.3121 (13)	0.719 (4)	0.6121 (14)	0.059 (7)*	
H50B	0.2951 (15)	0.526 (5)	0.6720 (16)	0.087 (10)*	
C51	0.20732 (10)	0.7172 (4)	0.59813 (10)	0.0356 (5)	
C52	0.18548 (12)	0.8875 (4)	0.54645 (12)	0.0459 (6)	
H52	0.2206 (13)	0.984 (4)	0.5387 (14)	0.069 (8)*	
C53	0.11413 (13)	0.9177 (4)	0.50569 (13)	0.0480 (6)	
H53	0.0951 (13)	1.038 (5)	0.4697 (15)	0.073 (8)*	
C54	0.06391 (11)	0.7793 (4)	0.51715 (13)	0.0403 (5)	
H54	0.0139 (12)	0.805 (4)	0.4887 (13)	0.045 (6)*	
C55	0.08516 (11)	0.6104 (4)	0.56866 (12)	0.0368 (5)	
H55	0.0505 (11)	0.512 (4)	0.5780 (12)	0.047 (6)*	
C56	0.15672 (11)	0.5793 (4)	0.60880 (11)	0.0360 (5)	
H56	0.1712 (11)	0.459 (4)	0.6452 (13)	0.045 (6)*	

### Atomic displacement parameters (Å^2^)

**Table:**

	*U*^11^	*U*^22^	*U*^33^	*U*^12^	*U*^13^	*U*^23^
N1	0.0250 (8)	0.0217 (8)	0.0205 (7)	−0.0004 (6)	0.0022 (6)	−0.0013 (6)
C11	0.0207 (8)	0.0171 (8)	0.0230 (8)	−0.0054 (7)	0.0027 (7)	−0.0011 (7)
C12	0.0282 (10)	0.0270 (10)	0.0288 (10)	−0.0068 (8)	0.0099 (8)	−0.0026 (8)
C13	0.0263 (10)	0.0276 (11)	0.0466 (12)	−0.0013 (9)	0.0121 (9)	−0.0051 (9)
C14	0.0260 (10)	0.0271 (11)	0.0423 (11)	0.0014 (9)	−0.0040 (9)	0.0022 (9)
C15	0.0354 (10)	0.0262 (10)	0.0248 (9)	−0.0038 (9)	0.0007 (8)	0.0010 (8)
C16	0.0273 (9)	0.0235 (9)	0.0230 (9)	−0.0036 (8)	0.0055 (7)	−0.0024 (8)
N2	0.0257 (8)	0.0187 (8)	0.0274 (8)	−0.0009 (6)	0.0028 (6)	−0.0006 (6)
C21	0.0316 (11)	0.0235 (10)	0.0345 (11)	−0.0049 (9)	0.0000 (9)	−0.0040 (9)
C3	0.0232 (9)	0.0269 (10)	0.0257 (9)	−0.0030 (8)	0.0045 (7)	0.0087 (8)
C31	0.0297 (11)	0.0307 (12)	0.0442 (13)	0.0013 (9)	0.0059 (9)	0.0048 (10)
C4	0.0241 (9)	0.0312 (10)	0.0200 (8)	−0.0064 (8)	0.0045 (7)	0.0041 (8)
C5	0.0277 (9)	0.0252 (10)	0.0184 (8)	−0.0062 (8)	0.0058 (7)	0.0022 (8)
O5	0.0351 (7)	0.0317 (8)	0.0270 (7)	−0.0036 (6)	0.0053 (6)	−0.0081 (6)
N41	0.0250 (8)	0.0400 (10)	0.0193 (7)	−0.0085 (7)	0.0040 (6)	0.0046 (7)
C42	0.0276 (10)	0.0412 (12)	0.0221 (9)	−0.0072 (9)	0.0070 (8)	0.0005 (9)
C43	0.0239 (9)	0.0498 (13)	0.0199 (8)	−0.0091 (9)	0.0073 (7)	−0.0022 (9)
C44	0.0275 (10)	0.0617 (15)	0.0317 (11)	−0.0018 (11)	0.0077 (9)	−0.0130 (11)
C45	0.0269 (10)	0.0733 (17)	0.0316 (11)	−0.0055 (11)	0.0079 (9)	−0.0244 (12)
C46	0.0208 (9)	0.0693 (15)	0.0210 (9)	−0.0081 (10)	0.0062 (7)	−0.0108 (10)
C47	0.0241 (10)	0.0583 (15)	0.0260 (9)	−0.0020 (10)	0.0075 (8)	−0.0067 (10)
C48	0.0290 (10)	0.0492 (14)	0.0243 (9)	−0.0082 (9)	0.0079 (8)	−0.0082 (9)
O49	0.0205 (7)	0.0846 (12)	0.0287 (7)	−0.0030 (7)	0.0046 (6)	−0.0241 (8)
C50	0.0243 (10)	0.089 (2)	0.0322 (11)	−0.0042 (12)	0.0047 (9)	−0.0266 (13)
C51	0.0268 (10)	0.0596 (14)	0.0192 (9)	−0.0038 (10)	0.0059 (8)	−0.0132 (10)
C52	0.0427 (13)	0.0631 (16)	0.0343 (11)	−0.0225 (12)	0.0159 (10)	−0.0083 (11)
C53	0.0551 (15)	0.0474 (14)	0.0331 (11)	−0.0063 (12)	0.0027 (10)	0.0021 (11)
C54	0.0274 (11)	0.0427 (13)	0.0414 (12)	−0.0007 (10)	−0.0018 (9)	−0.0124 (10)
C55	0.0294 (10)	0.0384 (12)	0.0412 (11)	−0.0091 (10)	0.0097 (9)	−0.0098 (10)
C56	0.0347 (11)	0.0425 (13)	0.0278 (10)	−0.0020 (10)	0.0059 (8)	−0.0056 (10)

### Geometric parameters (Å, º)

**Table:**

N1—C5	1.401 (2)	C42—C43	1.464 (2)
N1—N2	1.401 (2)	C42—H42	0.99 (2)
N1—C11	1.417 (2)	C43—C44	1.381 (3)
C11—C12	1.382 (2)	C43—C48	1.390 (3)
C11—C16	1.389 (2)	C44—C45	1.385 (3)
C12—C13	1.375 (3)	C44—H44	0.98 (2)
C12—H12	0.924 (18)	C45—C46	1.376 (3)
C13—C14	1.383 (3)	C45—H45	0.96 (2)
C13—H13	0.95 (2)	C46—O49	1.369 (2)
C14—C15	1.380 (3)	C46—C47	1.387 (3)
C14—H14	0.95 (2)	C47—C48	1.370 (3)
C15—C16	1.384 (3)	C47—H47	0.99 (2)
C15—H15	0.98 (2)	C48—H48	0.97 (2)
C16—H16	0.990 (18)	O49—C50	1.436 (3)
N2—C3	1.357 (2)	C50—C51	1.498 (3)
N2—C21	1.455 (2)	C50—H50A	0.95 (2)
C21—H21A	0.99 (2)	C50—H50B	1.09 (3)
C21—H21B	0.94 (2)	C51—C56	1.375 (3)
C21—H21C	0.97 (3)	C51—C52	1.378 (3)
C3—C4	1.363 (3)	C52—C53	1.379 (3)
C3—C31	1.479 (3)	C52—H52	0.96 (3)
C31—H31A	0.97 (2)	C53—C54	1.376 (3)
C31—H31B	0.99 (2)	C53—H53	0.98 (3)
C31—H31C	1.00 (3)	C54—C55	1.369 (3)
C4—N41	1.388 (2)	C54—H54	0.97 (2)
C4—C5	1.436 (3)	C55—C56	1.381 (3)
C5—O5	1.229 (2)	C55—H55	0.97 (2)
N41—C42	1.276 (2)	C56—H56	0.97 (2)
			
C5—N1—N2	109.57 (13)	N41—C42—C43	119.73 (19)
C5—N1—C11	124.71 (15)	N41—C42—H42	125.0 (12)
N2—N1—C11	119.21 (13)	C43—C42—H42	115.3 (12)
C12—C11—C16	120.85 (16)	C44—C43—C48	118.24 (18)
C12—C11—N1	118.70 (15)	C44—C43—C42	121.00 (19)
C16—C11—N1	120.45 (15)	C48—C43—C42	120.75 (18)
C13—C12—C11	119.18 (18)	C43—C44—C45	121.5 (2)
C13—C12—H12	120.2 (11)	C43—C44—H44	120.7 (13)
C11—C12—H12	120.6 (11)	C45—C44—H44	117.7 (13)
C12—C13—C14	120.70 (19)	C46—C45—C44	119.3 (2)
C12—C13—H13	119.1 (12)	C46—C45—H45	123.6 (13)
C14—C13—H13	120.2 (12)	C44—C45—H45	117.1 (13)
C15—C14—C13	119.85 (18)	O49—C46—C45	124.78 (19)
C15—C14—H14	119.2 (12)	O49—C46—C47	115.17 (18)
C13—C14—H14	120.9 (12)	C45—C46—C47	120.03 (18)
C14—C15—C16	120.26 (18)	C48—C47—C46	120.1 (2)
C14—C15—H15	122.5 (12)	C48—C47—H47	121.6 (12)
C16—C15—H15	117.2 (12)	C46—C47—H47	118.3 (12)
C15—C16—C11	119.12 (17)	C47—C48—C43	120.9 (2)
C15—C16—H16	120.9 (10)	C47—C48—H48	120.2 (12)
C11—C16—H16	120.0 (10)	C43—C48—H48	118.9 (12)
C3—N2—N1	106.76 (14)	C46—O49—C50	117.40 (16)
C3—N2—C21	123.27 (16)	O49—C50—C51	106.06 (18)
N1—N2—C21	117.24 (15)	O49—C50—H50A	106.2 (16)
N2—C21—H21A	109.6 (13)	C51—C50—H50A	108.4 (15)
N2—C21—H21B	108.9 (14)	O49—C50—H50B	108.6 (15)
H21A—C21—H21B	106.0 (18)	C51—C50—H50B	112.8 (15)
N2—C21—H21C	109.5 (16)	H50A—C50—H50B	114 (2)
H21A—C21—H21C	111 (2)	C56—C51—C52	118.83 (19)
H21B—C21—H21C	111 (2)	C56—C51—C50	119.8 (2)
N2—C3—C4	110.33 (16)	C52—C51—C50	121.3 (2)
N2—C3—C31	121.50 (17)	C51—C52—C53	120.5 (2)
C4—C3—C31	128.17 (17)	C51—C52—H52	119.2 (15)
C3—C31—H31A	111.9 (13)	C53—C52—H52	120.3 (15)
C3—C31—H31B	111.1 (14)	C54—C53—C52	120.1 (2)
H31A—C31—H31B	107.3 (19)	C54—C53—H53	115.4 (15)
C3—C31—H31C	109.6 (14)	C52—C53—H53	124.4 (16)
H31A—C31—H31C	109.3 (19)	C55—C54—C53	119.7 (2)
H31B—C31—H31C	107.5 (18)	C55—C54—H54	121.5 (13)
C3—C4—N41	121.31 (17)	C53—C54—H54	118.8 (13)
C3—C4—C5	108.33 (15)	C54—C55—C56	120.0 (2)
N41—C4—C5	130.32 (17)	C54—C55—H55	121.0 (13)
O5—C5—N1	123.76 (16)	C56—C55—H55	119.0 (13)
O5—C5—C4	131.83 (16)	C51—C56—C55	120.8 (2)
N1—C5—C4	104.39 (15)	C51—C56—H56	119.8 (13)
C42—N41—C4	122.30 (17)	C55—C56—H56	119.4 (13)
			
C5—N1—C11—C12	62.7 (2)	C3—C4—C5—N1	−1.39 (18)
N2—N1—C11—C12	−148.63 (16)	N41—C4—C5—N1	−178.76 (17)
C5—N1—C11—C16	−117.28 (19)	C3—C4—N41—C42	−177.14 (17)
N2—N1—C11—C16	31.4 (2)	C5—C4—N41—C42	−0.1 (3)
C16—C11—C12—C13	1.7 (3)	C4—N41—C42—C43	−179.59 (16)
N1—C11—C12—C13	−178.33 (17)	N41—C42—C43—C44	175.54 (18)
C11—C12—C13—C14	−2.5 (3)	N41—C42—C43—C48	−3.3 (3)
C12—C13—C14—C15	1.5 (3)	C48—C43—C44—C45	−0.1 (3)
C13—C14—C15—C16	0.4 (3)	C42—C43—C44—C45	−179.0 (2)
C14—C15—C16—C11	−1.2 (3)	C43—C44—C45—C46	0.0 (3)
C12—C11—C16—C15	0.1 (3)	C44—C45—C46—O49	−178.3 (2)
N1—C11—C16—C15	−179.87 (16)	C44—C45—C46—C47	−0.1 (3)
C5—N1—N2—C3	−8.15 (18)	O49—C46—C47—C48	178.61 (18)
C11—N1—N2—C3	−161.16 (15)	C45—C46—C47—C48	0.2 (3)
C5—N1—N2—C21	−151.23 (16)	C46—C47—C48—C43	−0.3 (3)
C11—N1—N2—C21	55.8 (2)	C44—C43—C48—C47	0.2 (3)
N1—N2—C3—C4	7.24 (19)	C42—C43—C48—C47	179.14 (18)
C21—N2—C3—C4	147.53 (18)	C45—C46—O49—C50	−5.7 (3)
N1—N2—C3—C31	−172.05 (16)	C47—C46—O49—C50	176.0 (2)
C21—N2—C3—C31	−31.8 (3)	C46—O49—C50—C51	−178.53 (18)
N2—C3—C4—N41	173.97 (15)	O49—C50—C51—C56	−103.0 (3)
C31—C3—C4—N41	−6.8 (3)	O49—C50—C51—C52	74.8 (3)
N2—C3—C4—C5	−3.7 (2)	C56—C51—C52—C53	−0.6 (3)
C31—C3—C4—C5	175.55 (18)	C50—C51—C52—C53	−178.4 (2)
N2—N1—C5—O5	−172.94 (15)	C51—C52—C53—C54	0.9 (3)
C11—N1—C5—O5	−21.7 (3)	C52—C53—C54—C55	−0.6 (3)
N2—N1—C5—C4	5.81 (17)	C53—C54—C55—C56	−0.1 (3)
C11—N1—C5—C4	157.01 (15)	C52—C51—C56—C55	−0.1 (3)
C3—C4—C5—O5	177.22 (18)	C50—C51—C56—C55	177.76 (19)
N41—C4—C5—O5	−0.2 (3)	C54—C55—C56—C51	0.4 (3)

### Hydrogen-bond geometry (Å, º)

**Table:**

*D*—H···*A*	*D*—H	H···*A*	*D*···*A*	*D*—H···*A*
C16—H16···O49^i^	0.990 (18)	2.486 (18)	3.470 (2)	172.6 (15)
C21—H21*C*···O5^ii^	0.97 (3)	2.58 (3)	3.352 (3)	137.3 (19)
C31—H31*A*···O5^ii^	0.97 (2)	2.65 (2)	3.517 (3)	149.1 (17)
C52—H52···O5^iii^	0.96 (3)	2.51 (3)	3.411 (3)	156 (2)

Symmetry codes: (i) −*x*+1, −*y*+2, −*z*+2; (ii) *x*, *y*+1, *z*; (iii) −*x*+1, *y*+1/2, −*z*+3/2.
